# Dedifferentiation of Mature Adipocytes and Their Future Potential for Regenerative Medicine Applications

**DOI:** 10.3390/biomedicines14010095

**Published:** 2026-01-02

**Authors:** Deniz Simal Bayulgen, Sheila Veronese, Andrea Sbarbati

**Affiliations:** Department of Neuroscience, Biomedicine, Movement Sciences, University of Verona, 37134 Verona, Italy; sheila.veronese@univr.it (S.V.); andrea.sbarbati@univr.it (A.S.)

**Keywords:** adipocyte dedifferentiation, DFAT cells, regenerative medicine, cancer-associated adipocytes, metabolic dysfunction

## Abstract

**Background/Objectives**: Mature adipocytes were previously regarded as terminally differentiated cells that are restricted to lipid storage. Recent studies have shown that they can dedifferentiate into fibroblast-like progenitor cells, termed dedifferentiated fat (DFAT) cells. These cells exhibit stem cell-like properties and multilineage potential, highlighting their promising role in regenerative medicine and disease pathology. This systematic review aims to explore and consolidate the evidence regarding mechanisms, culture methods, pathophysiological roles, and therapeutic potential of adipocyte dedifferentiation. **Methods**: A systematic review was conducted in PubMed using the terms “dedifferentiation”, “de-differentiation”, “transdifferentiation”, and related variants in combination with “adipocyte”. Studies were screened and selected according to the PRISMA 2020 guidelines. Non-English articles, non-full texts, and non-review papers were excluded. After duplicate removal and eligibility assessment, 53 studies were included. Further, these were classified into categories according to their abstracts. **Results**: The evidence from the included articles indicates that mature adipocytes can dedifferentiate both in vitro, via ceiling culture, and in vivo, yielding DFAT cells with proliferative and multilineage differentiation capacity. Dedifferentiation involves lipid droplet secretion (liposecretion) and is characterized by downregulation of adipogenic genes such as PPARG and C/EBPα, alongside upregulation of proliferation, stemness, and lineage-associated markers. Functionally, DFAT cells contribute positively to tissue regeneration and wound repair, but they can drive adverse outcomes such as fibrosis, insulin resistance, and tumor progression through signaling pathways, including Wnt/β-catenin and TGF-β. **Conclusions**: Mature adipocyte dedifferentiation marks a dynamic reprogramming mechanism with dual roles—beneficial in regenerative medicine and wound healing, yet detrimental in cancer and metabolic disease. Further research is required to identify in vivo regulators, establish definitive markers, and translate adipocyte plasticity into regenerative medicine applications.

## 1. Introduction

In the field of regenerative medicine, adult stem cells, particularly mesenchymal stem cells (MSCs), have received increasing attention due to their multipotency and immunomodulatory capabilities. Clinical evidence of their utility in various diseases is spreading, including graft-versus-host disease, inflammatory bowel disease, cardiovascular disorders, and neurological conditions [1]. Among the various MSC sources available, adipose tissue (AT) has attracted particular attention as an abundant, easily accessible, and ethically acceptable reservoir of MSCs.

Adipose tissue (AT) is the most extensive endocrine organ in the human body [2]. AT regulates energy storage and thermogenesis, including glucose and lipid metabolism, blood pressure homeostasis, inflammatory, and immunological responses [3]. AT is mainly categorized into two groups: white adipose tissue (WAT), whose main function is the storage of fatty acids for energy supply, and brown adipose tissue (BAT), which is abundant in mitochondria and functioning in thermogenesis [4]. A third type of adipocyte, known as the pink adipocyte, has recently been identified in mouse subcutaneous fat depots during pregnancy and lactation. Pink adipocytes are specialized mammary gland alveolar epithelial cells that are responsible for the production and secretion of milk. Recent evidence indicates that they originate from the transdifferentiation of subcutaneous WATs [5].

In the context of cellular plasticity, three distinct processes related to AT are often discussed: dedifferentiation, transdifferentiation, and cisdifferentiation. Dedifferentiation refers to the regression of a mature, specialized cell (e.g., a lipid-laden adipocyte) to a less specialized, progenitor-like state, regaining proliferative and multilineage potential [6]. In contrast, transdifferentiation (or direct reprogramming) describes the direct conversion of one differentiated cell type into another distinct lineage without an intermediate pluripotent state, such as the mentioned transition to pink adipocytes [6]. A third process, cisdifferentiation, is the conversion between closely related cell types within the same developmental lineage, requiring progression through specific stages. For instance, the genesis of oligodendrocytes from embryonic stem cells proceeds via obligatory neural ectoderm intermediates (cisdifferentiation), a pathway that is not accessible to cells from distant lineages, such as mesenchymal stem cells [7]. Analogous interconversions within the adipocyte lineage, such as those between white, brown, and beige adipocytes, also represent this form of lineage-restricted plasticity [8].

It is well known that AT is a great source of mesenchymal stem cells (MSCs) [9]. It has been reported that MSCs within the stromal vascular fraction (SVF) of subcutaneous adipose tissue show multilineage plasticity in vitro and in vivo. These pluripotent adult progenitor cells are given many different terms, such as adipocyte precursor cells, adipocytes, adipose-derived stem cells (ADSCs), and, ultimately, according to consensus, adipose-derived cells [10]. Additionally, adipose tissue includes mature adipocytes, which take part in the storage, utilization, and release of lipids [2]. In the past, the state of the mature fat cells with large lipid droplets (LDs) was considered an irreversible and terminal stage of differentiation. Recent research highlighted that mature adipocytes are capable of dedifferentiating into a population of proliferation-competent progeny cells termed adipofibroblasts or dedifferentiated fat (DFAT). This dedifferentiation is achieved using the ceiling culture method, which exploits the cells’ natural buoyancy. These cells share common characteristics with fibroblasts and their morphology; however, their functional properties are similar to those of pluripotent stem cells, especially their ability to differentiate into multiple cell lineages [11]. For this reason, DFAT cells hold great potential as a cell source for regenerative medicine applications, and numerous processing systems have been developed to extract and reuse these cells [12]. Up until now, DFAT cells have been isolated from various species, such as human, rat, mouse, and pig cells, and have demonstrated promising proliferation capabilities and multilineage differentiation. Recent research has demonstrated that human DFAT cells exhibit increased osteoblastic differentiation potential compared with ADSCs [13]. An additional study highlighted that mouse and human DFAT cells, originating from adipose tissue and lipoaspirate, can differentiate into vascular endothelial cells (ECs) both in vitro and in vivo [14]. Finally, another study found that DFAT cells, which were transplanted in rats and expressed the neuronal marker β-III tubulin, resulted in significant functional recovery from spinal cord injury-induced motor dysfunction [15]. These findings position DFAT cells, derived via dedifferentiation, as a promising and distinct cellular substrate for regenerative medicine.

**Figure 1:**
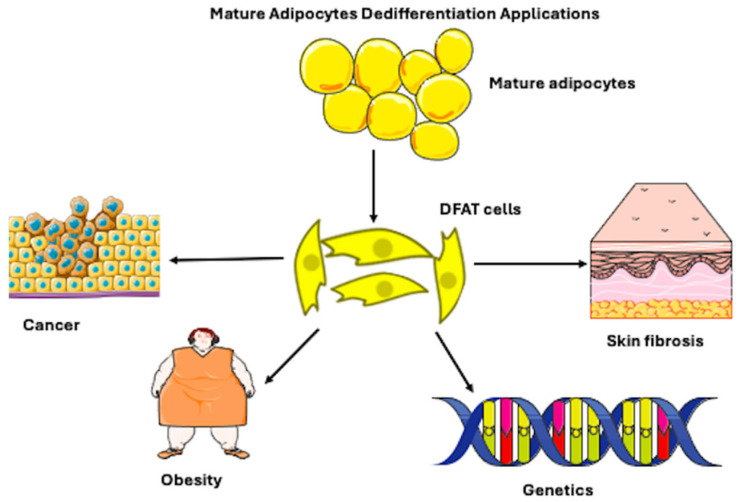
Figure illustrating that mature adipocytes, which are spherical cells, possess the ability to differentiate into fibroblast-like-shaped, dedifferentiated fat (DFAT) cells. The mechanism of dedifferentiation may be used in regenerative applications for cancer, obesity, genetic, and skin fibrosis pathologies. However, it can be present in physiological conditions. Adapted from Servier Medical Art (https://smart.servier.com), licensed under CC BY 4.0 (https://creativecommons.org/licenses/by/4.0/), and NIAID Visual & Medical Arts. (10/7/2024). Fibroblast. NIAID NIH BIOART Source. bioart.niaid.nih.gov/bioart/153.

Adipocyte dedifferentiation involves the reconstruction of their morphological structure from a large, unilocular appearance to a fibroblast-like state. This process leads to a reduction in the size and number of adipocytes. In turn, this may open new perspectives in addressing the issue of localized fat deposition in obesity. Furthermore, DFAT cells seem to be involved in various pathologies, such as breast and pancreatic cancer, and seem to be related to tumor progression [16]. Finally, they are not limited only to in vitro induction [17]. Hence, exact regulation of adipocyte dedifferentiation has gained attention in terms of disease therapies in clinics and regenerative medicine applications (Figure 1). Therefore, the systematic exploration of adipocyte dedifferentiation represents a critical junction between cell biology and translational regenerative medicine. This systematic review has three principal aims: First, it aims to synthesize the current evidence on the molecular mechanisms, culture techniques, and defining characteristics of mature adipocyte dedifferentiation and the resulting DFAT cells. Second, it aims to elucidate the dual physiological roles of these cells by detailing their beneficial functions in tissue repair and their detrimental contributions to metabolic disease, fibrosis, and cancer progression. Finally, it intends to critically evaluate the therapeutic potential and inherent risks of harnessing adipocyte dedifferentiation for regenerative medicine, thereby identifying crucial knowledge gaps for future research.

## 2. Methodology

The detailed protocol for this systematic review was registered on the Open Science Framework prior to conducting the literature search (Registration: https://osf.io/9gr25: accessed on 2 December 2025). We conducted a systematic literature review of the articles published up to July 2025, using the PubMed database (according to Table S1: PRISMA 2020 Checklist). The names of the six different categories of adipocyte differentiation were used for the search (“Dedifferentiation”, “De-differentiation”, “Cisdifferentiation”, “Cis-differentiation”, “Transdifferentiation”, “Trans-differentiation”), together with the term “adipocyte”, with each search being performed independently before merging all the results.

During screening, only studies that addressed adipocyte differentiation, dedifferentiation, transdifferentiation, or other forms of adipocyte fat conversion in adipocytes, preadipocytes, or adipose-derived cells were considered. The inclusion criteria for the identified articles were as follows: only review articles, written in English, with the full text available, and discussing biological aspects.

Exclusion criteria were articles that were not related to adipocyte biology and non-research articles, such as editorials or conference abstracts.

Finally, duplicate records were removed.

**Figure 2:**
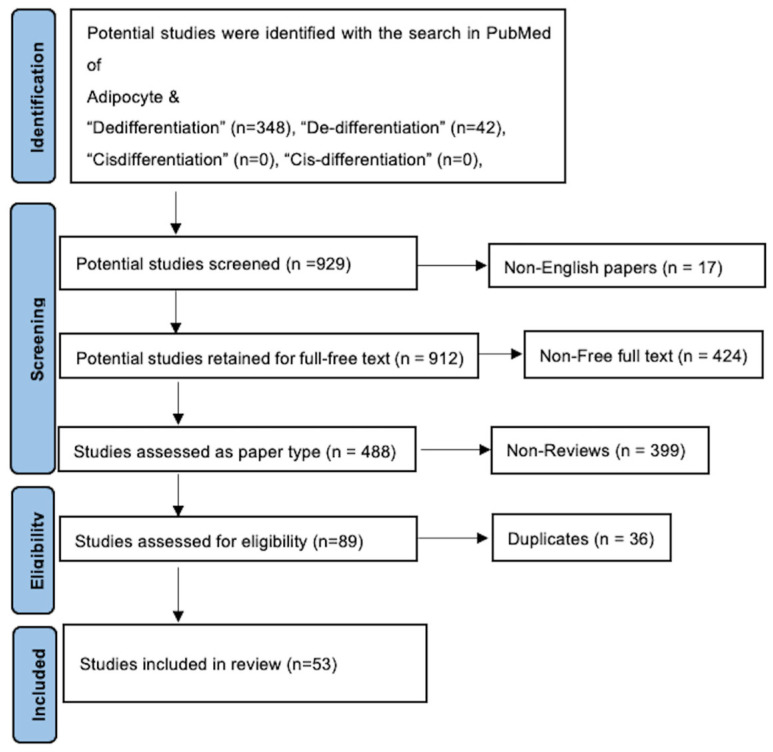
PRISMA flow diagram. After utilization of inclusion–exclusion criteria, the total number of included studies was 53.

In summary, review articles that met the eligibility criteria were included, along with studies cited in these reviews that were relevant. No formal biases or clinical scoring tools were used, as the focus was on biological mechanisms and cell fate processes. The stepwise selection is illustrated in the PRISMA flowchart [18] in Figure 2.

## 3. Results

The systematic search yielded 89 review articles. Following the removal of 36 duplicates, 53 unique review articles were screened based on the predefined eligibility criteria. During the title and abstract screening, articles were excluded as “non-related” if their primary focus was not the dedifferentiation or direct fate conversion of mature adipocytes. This specifically permitted us to exclude three types of studies: (1) studies related to the differentiation of stem/progenitor cells into adipocytes (adipogenesis without evidence of mature cell reversal); (2) studies concerning the general biology of adipose-derived stem/stromal cells that were unrelated to a mature adipocyte origin; and (3) articles related to transdifferentiation into unrelated lineages without a dedifferentiated intermediary. Consequently, 53 articles met all the eligibility criteria and were included for synthesis. As this is a systematic review designed to map the conceptual landscape and identify themes, a formal risk-of-bias assessment was not performed.

The included research comprises a range of study designs, including primary experimental research (both in vitro and in vivo), narrative reviews, and mechanistic studies, reflecting the broad methodological approaches used in this field. A thematic analysis of the 53 articles was performed. Most of the literature was divided into two overarching categories based on the primary focus of the plasticity described: dedifferentiation and transdifferentiation articles.

**Figure 3:**
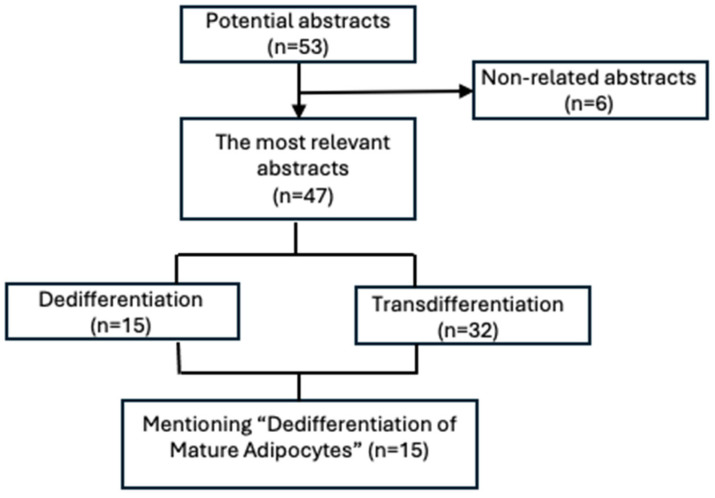
Diagram showing the further categorization of articles into two subheadings: dedifferentiation and transdifferentiation. Subsequently, the articles that were most related to the “Dedifferentiation of Mature Adipocytes” theme were classified into various categories to analyze dedifferentiation in different physiological and pathological conditions.

Fifteen articles were identified as specifically and directly investigating “Dedifferentiation of Mature Adipocytes.” These 15 papers were further subcategorized based on their research context. Articles were assigned to a single, primary subcategory according to the central focus of the paper, which was determined by analyzing the study’s aims, model systems, and key conclusions. The five subcategories and their defining criteria were as follows: (1) cancer—studies examining dedifferentiation in the context of tumor biology (e.g., tumor microenvironment, cancer-associated adipocytes); (2) morphology/culture—studies with a primary emphasis on characterizing the in vitro process, including morphological changes and culture conditions of DFAT cells; (3) obesity/metabolic syndrome—studies investigating dedifferentiation within the pathophysiological framework of metabolic disease or adipose tissue dysfunction; (4) genetics—studies focused on elucidating the specific molecular drivers, signaling pathways, or gene expression programs regulating the dedifferentiation switch; and (5) myofibroblast formation—studies where the terminal fate of dedifferentiated cells was explicitly linked to a fibroblast/myofibroblast lineage in contexts of fibrosis or scar repair. To handle studies that logically span multiple themes (e.g., a study on cancer that also explores underlying genetic pathways), a primary categorization was made based on the dominant research question. These thematic overlaps are explicitly acknowledged within the narrative synthesis, listing a study’s primary category and noting any significant secondary themes to ensure transparency. This categorization is presented in Figure 3 and Table 1.

### 3.1. Mechanisms of Mature Adipocyte Dedifferentiation

Adipocytes are typically regarded as being at the end of their differentiation process, and they are considered stationary, since they are no longer able to proliferate. However, according to new research, mature adipocytes may undergo a process known as transdifferentiation that allows them to reversibly alter their phenotype and change into cells with a different morphology and physiology [13,14]. It is not clear if this process involves the gradual dedifferentiation of the primary cell into an intermediate cell type that is capable of differentiating into a new cell lineage, or if it directly transforms one cell type into another. It is also poorly understood how mature adipocytes dedifferentiate in vivo [19]. The adipocyte dedifferentiation process was first revealed by Sugihara and colleagues in 1986. They found that through ceiling culture, mature adipocytes transform into fibroblast-like cells expressing a stem cell signature [20]. Further research by these authors explored how those mature adipocytes could enter the cell cycle and divide. This demonstrated that they are not cells in a quiescent state [11].

The dedifferentiation of adipocytes has been better understood thanks to research conducted over the past ten years. Maurizi et al. [21] initially recognized the phenomenon characterizing the mature adipocyte dedifferentiation in ceiling culture and called it “liposecretion”. This corresponds to the secretion of a large intracellular lipid droplet coated by a three-layered membrane [21]. It appears that the presence of the cyclin-dependent kinase 1 inhibitor RO-3306 prevents this phenomenon [22]. Moreover, research shows that insulin has a negative regulatory effect on adipocyte dedifferentiation [23].

Nonetheless, adipocyte dedifferentiation is not merely a spontaneous event in ceiling culture; it can be actively induced or accelerated by specific experimental manipulations that mimic pathological or stressful microenvironments. These inducers can be broadly categorized: First, physical and osmotic stress, such as exposure to high osmotic pressure (~400 mosmol/L) or mechanical compression, mimics the solid stress of a growing tumor and can induce rapid lipid loss and fibroblast-like morphology, often via activation of the Wnt/β-catenin pathway [24]. Second, the blockade of adipogenic hormones is a potent trigger; the removal of insulin/IGF-1 receptor inhibitor OSI-906 (Litsitinib) initiates and accelerates dedifferentiation [23]. Third, exposure to inflammatory and profibrotic factors that are present in the disease context, such as TGF-β1, TNF-α, or oncostatin M (OSM), or found in inflammatory zone 1 (FIZZ1), can drive the process [17]. Other signaling molecules, such as Wnt3a, which antagonizes adipogenesis, and physical stimuli, like cold exposure (e.g., 10 °C), have also been demonstrated to be effective inducers [17]. This repertoire of experimental triggers highlights that dedifferentiation is a responsive plasticity program, activated when mature adipocytes encounter specific biochemical and biophysical challenges. Overall, it has been hypothesized that dedifferentiation could be a form of cellular reprogramming in which mature adipocytes develop multipotency and lose their functional phenotype [25].

An additional study enabled a better description of adipocyte dedifferentiation. The study of liposecretion was advanced by using live-cell time-lapse microscopy, which highlighted that mature adipocytes secrete, at high speed, their intracellular lipid droplets, becoming fibroblast-like cells [26]. Finally, a post-adipocytic phase has been studied through transmission and scanning electron microscopy (TEM and SEM) [27].

### 3.2. Morphology and Culture Technique of Dedifferentiated Fat (DFAT) Cells

**Figure 4:**
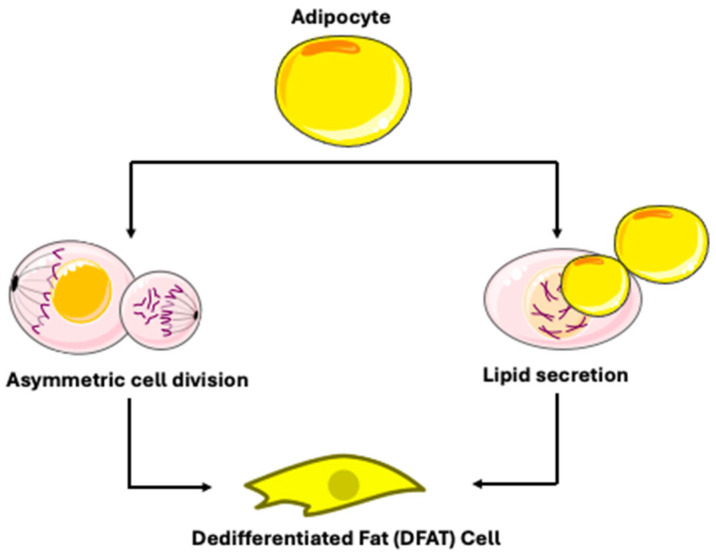
Mechanisms of dedifferentiated fat cell production. Adapted from Servier Medical Art (https://smart.servier.com), licensed under CC BY 4.0 (https://creativecommons.org/licenses/by/4.0/).

DFAT cells were produced using the ceiling culture method [28]. This approach includes cultivating fat cells in a bottle filled with medium, where buoyancy causes the fat cells to adhere to the top of the bottle [17]. When the culture flask was flipped and filled with culture medium, mature adipocytes progressively flattened and lost their spherical morphology [28]. Microscopic analysis of DFAT cells at various time points demonstrated a progressive loss of lipid-laden material, starting on day 1, continuing through days 3 and 7, and culminating in total evacuation by day 14 [17]. Within 1–2 weeks, lipid droplets inside the adipocytes completely vanished, and the cell shape gradually changed from round to long spindle, leading to a fibroblast-like cell morphology [28]. Previous research had already shown that adipocyte dedifferentiation is not attributable to a gradual loss of lipids (e.g., lipidolysis), but rather to a phenomenon referred to as “lipid secretion” [17], characterized by the rapid secretion of a large intracellular lipid droplet coated by a three-layered membrane [17,22,29]. Ultrastructural analysis revealed that this process is accompanied by the development of secretory organelles, including a hypertrophic Golgi complex and accumulated rough endoplasmic reticulum [17]. Live-imaging using specialized ceiling culture chips has shown that this lipid droplet expulsion involves dynamic actin cytoskeleton remodeling and can occur rapidly [30]. Building on the progress of ceiling culture, researchers explored the production of fibroblast-like cells from mature adipocytes through a process of asymmetric division. The methodologies used were Bromodeoxyuridine (BrdU) detection and time-lapse fluorescence microscopy [17]. This division process produces one lipid-filled adipocyte and one lipid-free subcell. Following this, these lipid-free cells underwent dedifferentiation, ultimately resulting in the formation of DFAT cells. This characteristic of asymmetric division has also been seen during the dedifferentiation of adult adipocytes in cattle and pigs. However, a key contrast is evident in experimental models and observations: while liposecretion is frequently reported in human and rodent adipocyte cultures [17,22], direct observation of asymmetric division in modern, lineage-traced models is less common. For instance, a study of an Adiponectin-Cre recombinase mouse model for unambiguous tracking of mature adipocytes reported observing liposecretion but did not observe asymmetrical division [31]. Thus, the dedifferentiation of mature adipocytes seems to result from two different phenomena: lipid secretion and asymmetric division (Figure 4) [17]. Current evidence suggests that these mechanisms may represent context-dependent alternatives rather than universally coexisting pathways. The dominant mechanism appears to be influenced by experimental conditions and possibly species-specific factors. Liposecretion is identified as a primary and frequently observed mechanism in ceiling culture and has been shown to be dependent on cell cycle regulators like CDK1 [22]. In contrast, the evidence for asymmetric division as a widespread mechanism is less definitive in contemporary, rigorously controlled studies using lineage-tracing techniques [31].

### 3.3. Physiological and Pathophysiological Roles of Adipocyte Dedifferentiation

Recent studies indicate that adipocytes could dedifferentiate into pluripotent progenitor cells in vivo under both healthy and pathological conditions [17].

Lineage tracing has shown that, in the mouse, “pink” adipocytes of the mammary gland can differentiate into mammary epithelial cells during lactation and subsequently revert to adipocytes during involution [32]. However, these findings have been debated by other studies [33], suggesting that it is adipocyte progenitors that transition into epithelial cells.

Adipocyte dedifferentiation has been associated with various cancers, including breast cancer [34] and colon cancer [35]; one study suggests a potential therapeutic efficacy of Peroxisome Proliferator-Activated Receptor Gamma (PPARG) agonist treatment to revert breast cancer cells into adipocytes, while a second study described a mesenchymal transition process of the peritumoral adipose tissue that could be a valid target for tumor therapy [35].

Finally, dedifferentiated white adipocytes serve as a source of stem cells for the repair of cardiac tissue [36] and spinal cord injuries [15]. Adipocytes in dermal white adipose tissue can differentiate into myofibroblasts and contribute to wound healing [37].

#### In Vivo Evidence for Adipocyte Plasticity

A critical synthesis of in vivo studies reveals a consistent demonstration of adipocyte plasticity across various physiological and pathological contexts. Moreover, it highlights important methodological and interpretative distinctions. In murine models of mammary gland remodeling, lineage tracing using inducible, adipocyte-specific Cre systems (e.g., Adipoq-CreERT2) has provided robust evidence that mature adipocytes dedifferentiate into a proliferative, fibroblast-like state during pregnancy and reliably redifferentiate post-lactation, defining a cyclical and fully reversible plasticity that is essential for tissue homeostasis [38]. In contrast, studies on dermal wound healing and fibrosis utilize complementary strategies, including intersectional fate mapping (e.g., Adipoq-Cre; Pdgfra-reporter) and exposure to profibrotic challenges like bleomycin [39]. These models show that dermal adipocytes downregulate adipogenic markers and adopt a myofibroblast phenotype, driven by signaling pathways such as PDGF and TGF-β; however, this process often contributes to a maladaptive, progressive fibrotic response rather than a reversible cycle [40]. In the context of obesity and metabolic disease, investigating both mice and humans and leveraging techniques like single-nucleus RNA sequencing identifies a persistent “obesogenic downregulation” of metabolic genes and a shift toward a pro-fibrotic state that predisposes to metabolic dysfunction [41]. A common molecular pattern across these models is the concerted downregulation of core adipogenic transcription factors (e.g., PPARG, C/EBPα) and lipid droplet proteins (e.g., Plin1) [42,43]. However, a key discrepancy lies in the functional outcome and precision fate mapping. The highly specific, inducible genetic tracing in mammary studies confirms direct and reversible fate change in mature adipocytes [38]. In other settings, evidence can be more indirect, and the plasticity often appears unidirectional and pathological, contributing to fibrosis or metabolic dysregulation [40].

### 3.4. Implications in Metabolic Dysregulation

The process of dedifferentiation involves mature adipocytes losing their characteristic lipid droplets and reverting to a more primitive, fibroblast-like state [17]. This loss of lipid storage capacity during dedifferentiation is a key phenomenon that could be linked to systemic metabolic dysregulation. Accordingly, Ono et al. [44] stated that dedifferentiation led to the downregulation of 308 genes, which were involved in lipid metabolism of Adipocyte Q (ADIPOQ), Lipase Hormone-Sensitive (LIPE), Pyruvate Dehydrogenase Kinase 4 (PDK4), Lipoprotein Lipase (LPL), Fatty Acid Synthase (FASN), PPRAG, and Fatty Acid-Binding Protein 4 (FABP4) [25].

In contrast, gene expression analysis in the adipose tissue depots revealed a significant reduction in lipogenic and adipogenic pathways, including C CAAT/Enhancer-Binding Protein Alpha (C/EBPα), C CAAT/Enhancer-Binding Protein Beta (C/EBPβ), and Sterol Regulatory Element-Binding Transcription Factor 1 (SREBF1). Supporting this, studies show that mice that overexpress the activated form of the Notch1 receptor specifically in mature adipocytes have a significantly lower body fat mass than controls [45], while conversely, haploinsufficiency of Notch1 accelerates adipogenesis and fat accumulation [46]. Therefore, the functional failure of adipose tissue, characterized by this absence of proper storage space for triacylglycerols due to impaired adipocyte function, causes ectopic lipid accumulation and “lipotoxicity” [47]. This condition, in turn, leads to severe insulin resistance, mirroring the metabolic disturbances seen in the lipodystrophic state [48].

### 3.5. Roles in Cancer, Tissue Repair, and Fibrosis

Dedifferentiated adipocytes have been recognized in pathologies including skin fibrosis, wound healing, and cancer using lineage tracing techniques on mouse models [34,49,50]. There is a growing body of evidence regarding the dedifferentiation of tumor-associated adipocytes within the tumor microenvironment of various cancer typologies, such as breast cancer [34], pancreatic cancer [16], nasopharyngeal carcinoma [26], and cancer of the colon [35], as well as liposarcoma (LPS) [51]. Tumor-associated adipocytes have been reported to contribute to the aggressiveness of tumor cells. For this reason, they have been referred to as cancer-associated adipocytes (CAAs) [52]. Of particular interest is the study by Zoico et al. [16], who proved that 3T3-L1 adipocytes—a widely used mouse cell model for mature fat cells—lose fat droplets and differentiate into CAAs when cocultured with pancreatic cancer cells (Mia-PaCa2, a human pancreatic carcinoma cell line) or melanoma cells. This explains why CAAs may contribute to the malignant characteristics of tumors [16]. This transformation of tumor-associated adipocytes into CAAs is driven by specific molecular reprogramming, initiated within the tumor microenvironment. Cancer cell-derived signals, including cytokines such as, TGF-β1, TNF-α, and interleukin 11 (IL-11), activate pathways such as NF-κB, which directly suppress the master adipogenic regulators PPARG and C/EBPα, thereby inducing mature adipocytes to exit their differentiated state [53,54,55]. Several signaling cascades have been identified as mediators of this dedifferentiation. In ovarian and breast cancers, tumor-derived Wnt ligands (e.g., Wnt3a, Wnt5a) activate the canonical Wnt/β-catenin pathway, stabilizing β-catenin to transcriptionally repress adipogenic genes [53,56]. Concurrently, the activation of the TGF-β1/SMAD3 pathway upregulates Tribbles homolog 3 (TRIB3), which inhibits C/EBPβ phosphorylation, further entrenching the dedifferentiated phenotype [57]. Additionally, emerging evidence implicates the Hippo pathway effectors YAP/TAZ, whose activation in dysfunctional adipocytes promotes a CAA-like, fibroblastic transformation [58]. Functionally, CAAs are active architects of a pro-tumorigenic niche. They undergo extensive metabolic reprogramming, enhancing lipolysis to release free fatty acids and lactate, which cause cancer cell growth and metastasis through β-oxidation [17,58,59]. Furthermore, CAAs secrete a distinct “adipokine storm”—including increased levels of LEP, IL-6, CCL2, and visfatin, as well as reduced ADIPOQ—which remodels the ECM, promotes angiogenesis, and contributes to an immunosuppressive tumor microenvironment by influencing macrophage polarization and T-cell function [53]. This bidirectional crosstalk establishes a vicious cycle where cancer cells induce adipocyte dedifferentiation, and the resulting CAAs, in turn, provide metabolic, inflammatory, and structural support that drives tumor progression, aggressiveness, and therapy resistance [54,58].

Bochet and coworkers revealed that CAAs aggregate in mouse mammary cancer tissue and contribute to the desmoplastic reaction detected around the tumor [45]. In the pathogenesis of cutaneous fibrosis, the mechanism of adipocyte dedifferentiation into fibroblast-like cells is regulated by the protein Transforming Growth Factor Beta 1 (TGFβ1), which plays a crucial role in regulating cell growth and differentiation [60]. In fact, in an Adiponectin promoter-driven Cre recombinase (Adipoq-Cre) transgenic mouse model of bleomycin-induced skin fibrosis, triple-positive “transition cells” expressing the adipocyte marker tdTomato perilipin, and the myofibroblast marker alpha-smooth muscle actin (α-SMA) was detected in the dermal white adipose tissue. However, perilipin expression was lost during fibrosis accumulation. This corresponds to the transformation of the adipocytes into myofibroblasts [60].

Finally, it has been demonstrated that during wound healing, severe lipolysis reduces the cells’ stored lipids, allowing dermal fat cells to dedifferentiate into fibroblasts, migrate to the wound bed, and then redifferentiate into fat cells, thus accelerating wound healing [50]. Furthermore, fibroblasts produced by adipocyte dedifferentiation might redifferentiate into adipocytes and may facilitate the reprogramming of disordered fibrous tissue in scars into adipose tissue, thus improving scar morphology [61,62].

### 3.6. Molecular Pathways and Phenotypic Changes in Adipocyte Dedifferentiation

DFAT cells encounter considerable alterations in gene expression during their transformation to a fibroblast-like phenotype [44]. In line with this, past studies observed significant morphological alterations during the dedifferentiation process. Cells lose their round appearance and show an elongated shape as they release their lipid content. On day 12 of the process, cells possess a fibroblastic shape. Firstly, some studies found significant changes linked to the lipogenic and adipogenic pathways of mature adipocytes in DFAT cells. The expressions of lipoprotein lipase (LPL), leptin (LEP), glucose transporter type 4 (GLUT4), peroxisome proliferator-activated receptor gamma (PPARG), and CCAAT/enhancer binding protein alpha (CEBPα) were either completely absent or drastically reduced. PPARG and CEBPA, and adiponectin (ADIPOQ) gene expression, were much lower in DFAT cells than in mature adipocytes [63].

Secondly, the expression of stem cell markers is increased in DFAT cells [21]. This implies the activation of genes related to embryonic stem cells and reprogramming, accompanied by the restoration of a hypomethylation pattern resembling that of stem cells [55,63]. Consistently, DFAT cells have been shown to express a panel of pluripotency-associated factors, including Oct4, Sox2, Nanog, c-Myc, and the surface antigens SSEA-1, CD31, and CD105, along with alkaline phosphatase and telomerase activity, confirming acquisition of a stem-like molecular signature that is distinct from that of ADSCs [64].

Thirdly, genes associated with the cell cycle and proliferation are upregulated in DFAT cells. These upregulated genes are involved in processes such as mitosis, cell division, and cell cycle progression [44]. In vitro experiments have demonstrated that DFAT cells showed similar proliferation capabilities to those of ADSCs [65].

Fourthly, genes that are relevant to lineage differentiation are upregulated [44]. These upregulated genes include all three germ layers, and this confirms that DFAT cells may differentiate into multiple cell lineages [66].

The fifth category is the overexpression of genes associated with cellular processes. Genes that are involved in activities such as cell motility, cell migration, tissue development, cell growth, cell proliferation, cell morphogenesis, and shape changes are upregulated during adipocyte dedifferentiation [44]. The key molecular markers and regulatory factors of the different stages of adipocyte dedifferentiation are summarized in Table 2.

Additionally, some studies highlighted that the dedifferentiation process significantly increased the expression of genes encoding proteins that are involved in extracellular matrix (ECM) remodeling, such as matrix-metalloproteinase 1 (MMP1), fibroblast-activated protein (FAP), dipeptidyl peptidase IV (DPP4), and transforming growth factor β1 (TGFβ1). Altogether, these transcriptional and ECM changes are orchestrated by converging PPARG, Notch, Wnt/β-catenin, and TGF-β/SMAD signaling, which progressively drive the transition from a lipid-laden, insulin-sensitive adipocyte toward a proliferative, fibroblast-like, and matrix-remodeling phenotype during dedifferentiation [67,68]. Multiple studies have found that key regulators of adipogenesis and adipodifferentiation, including PPARG and C/EBPα, are downregulated. This indicates a return to predifferentiation gene expression [44,69]. Through specific induction conditions, PPARG and C/EBPα may reactivate, which can lead to cell differentiation into adipocytes [70,71].

In terms of fatty acid oxidation, adipocyte dedifferentiation has been proven in a mouse model that administered an adenovirus expressing the Notch1 intracellular domain (Ad/N1ICD) [72]. Lipidomics analysis demonstrated the downregulation of fatty acid oxidation, lipid uptake, and oxidation pathways in Ad/N1ICD adipose tissue [44].

**Table 2:** Key molecular markers and regulatory factors across stages of adipocyte dedifferentiation.

Dedifferentiation Stage	Molecular Markers	Transcriptional Factors	Selected References and Context
1. Mature Adipocyte (Initial State)	White Adipocytes: High levels of PLIN1 [73], FABP4 [74], ADIPOQ [75], LEP [37]. Depot-specific markers: TCF21 [76] (visceral), HOXC8/9 [77] (subcutaneous).	Master transcriptional regulators of the mature state: PPARG, C/EBPα [78]. Their downregulation is a hallmark of dedifferentiation initiation [69].	Markers define the starting point. Their loss is tracked during dedifferentiation [69].
	Brown Adipocyte: High UCP1 [79], PRDM16 [80], CIDEA [81], ZIC1 [82].		
	Beige Adipocyte: Inducible UCP1 [79], TMEM26 [83], CD137 [84], TBX1 [57].		
2. Early/Initiating Phase	Downregulation: Rapid decrease in mature adipocyte transcripts (PPARG, C/EBPα, ADIPOQ, LPL) [69].	Inducing Stimuli: Ceiling culture technique; Tumor microenvironment (high osmotic pressure, TNF-α); Wnt3a, TGF-β1 signaling; Cold stimulation [17].	This phase involves exiting the mature state. In vivo examples include pregnancy/lactation in mammary glands, and dermal adipocytes during hair follicle regression [17].
	Upregulation: Early stress/injury response signals and matrix remodeling genes (FAP, DPP4) [69].	Cellular Process: Activation of “liposecretion”-the secretion of membrane-wrapped lipid droplets, dependent in processes like CDK1 activity [22].	
3. Intermediate/Transitional State	Loss: Lipid droplet proteins (e.g., PLIN1) [17].	Pathway Driving Change: ECM remodeling (sustained FAP, DPP4, MMP1, TGF-β1) [69]; Inflammatory cytokine secretion (IL-6, IL-8) [69]; Modulation of autophagy and cell-cycle re-entry [22].	Cell adopts a fibroblast-like morphology. This state is observed in pathological conditions, such as cancer-associated adipocytes and skin wound healing [17].
	Gain: Re-expression of progenitor/pre-adipocyte markers (e.g., PDGFRA, VIM, COL1A1). Cells may co-express markers of both fates (e.g., perilipin+ and α-SMA+ “transition cells” in fibrosis) [17].		
4. Dedifferentiated Fat (DFAT) Cell/Progenitor-like State	Marker Profile: Fibroblast-like morphology; minimal lipid content; Expression of mesenchymal/stem cell markers (e.g., CD90, CD105), absence of hematopoietic marker CD45 [85]. Multilineage potential (adipogenic, osteogenic, chondrogenic) [86].	Maintained Regulators: Activities that suppress redifferentiation and maintain proliferative capacity [86].	DFAT cells are considered multipotent and are a focus for regenerative medicine. They can be derived from white, brown, and beige adipocytes [86].

ADIPOQ (Adiponectin); α-SMA (Actin Alpha 2, Smooth Muscle); C/EBPα (CCAAT/Enhancer Binding Protein Alpha); CD137 (Tumor Necrosis Factor Receptor Superfamily Member 9); CD45 (Protein Tyrosine Phosphatase Receptor Type C); CD90 (Thy-1 Cell Surface Antigen); CD105 (Endoglin); CDK1 (Cyclin Dependent Kinase 1); CIDEA (Cell Death Inducing DFFA Like Effector A); COL1A1 (Collagen Type I Alpha 1 Chain); DPP4 (Dipeptidyl Peptidase 4); FABP4 (Fatty Acid Binding Protein 4); FAP (Fibroblast Activation Protein Alpha); HOXC8 (Homeobox C8); HOXC9 (Homeobox C9); IL-6 (Interleukin 6); IL-8 (Interleukin 8); LEP (Leptin); LPL (Lipoprotein Lipase); MMP1 (Matrix Metallopeptidase 1); PDGFRA (Platelet Derived Growth Factor Receptor Alpha); PLIN1 (Perilipin 1); PPARG (Peroxisome Proliferator Activated Receptor Gamma); PRDM16 (PR/SET Domain 16); TBX1 (T-Box Transcription Factor 1); TCF21 (Transcription Factor 21); TGF-β1 (Transforming Growth Factor Beta 1); TMEM26 (Transmembrane Protein 26); TNF-α (Tumor Necrosis Factor Alpha); UCP1 (Uncoupling Protein 1); VIM (Vimentin); ZIC1 (Zic Family Member 1).

### 3.7. Molecular Crosstalk of Key Signaling Pathways in Adipocyte Dedifferentiation

The dedifferentiation of mature adipocytes is regulated by a series of interacting signaling pathways that converge to suppress the master adipogenic regulator PPARG, thereby reversing the terminally differentiated state [87,88]. The specific roles and crosstalk of the principal pathways are as follows:

- PPARG Downregulation is a Central Event: The PPARG is the master transcriptional regulator and essential for establishing and maintaining the mature adipocyte phenotype, including lipid storage and insulin sensitivity. Its downregulation is a hallmark and prerequisite for dedifferentiation to initiate [88].
- Canonical Wnt/β-catenin Pathway as a Primary Repressor: Activation of the canonical Wnt/β-catenin pathway is a potent inhibitor of adipogenesis and a promising driver of dedifferentiation. In the active state, stabilized β-catenin translocates to the nucleus, where it complexes with T-cell factor/lymphoid enhancer-binding factor (TCF/LEF) proteins to transcriptionally repress PPARG gene expression [87,89]. Furthermore, Wnt/β-catenin signaling can inhibit PPARG transactivation via histone-modifying enzymes. In the context of cancer, tumor-derived Wnt ligands (e.g., Wnt3a, Wnt5a) activate this pathway in adjacent adipocytes, directly suppressing PPARG and C/EBPα to induce a dedifferentiated, cancer-associated adipocyte (CAA) phenotype. This pathway’s activity is also upregulated by biophysical stressors such as tissue compression, linking mechanical cues to dedifferentiation [53].
- TGF-β/SMAD Pathway in Fibroblastic Transformation: The TGF-β pathway promotes dedifferentiation towards a fibroblastic or myofibroblastic state [90]. Upon TGF-β1 binding, the receptor SMADs (SMAD2/3) are phosphorylated, complex with SMAD4, and translocate to the nucleus. This signaling directly suppresses adipogenic transcription factors and upregulates the encoding of ECM components (e.g., collagens, fibronectin) and the myofibroblast marker α-SMA. In pathologies such as fibrosis and cancer, TGF-β1 is a major driver of the adipocyte-to-myofibroblast transition [90].
- Notch Signaling Modulates Plasticity: The Notch pathway exerts context-dependent effects on adipocyte plasticity. Activation of Notch signaling, such as through the Notch1 intracellular domain (N1ICD), inhibits adipogenic differentiation by repressing PPARG and C/EBPα expression [91]. Consequently, the gain of Notch function in adipocytes promotes dedifferentiation and is associated with lipodystrophy, while its inhibition can accelerate adipogenesis. Notch interacts with other pathways; for instance, it can inhibit Wnt/β-catenin signaling, creating a complex regulatory network [91].

## 4. Discussion

This review synthesizes current findings on the dedifferentiation of mature adipocytes, exposing it as a dynamic and diverse process with substantial consequences across physiology and disease. Mature adipocytes may revert to progenitor-like cells termed dedifferentiated fat (cells which exhibit stem cell features and multilineage differentiation potential, both in vitro and in vivo), demonstrating a conserved plasticity across species. The debate demonstrates that adipocyte dedifferentiation is not only a reversal of differentiation but a complicated reprogramming process, leading to the creation of multipotent DFAT cells. Gene expression studies have demonstrated that significant adipogenic genes such as PPARG and C/EBPα are downregulated early during dedifferentiation, whereas indicators of proliferation, stemness, and migration are elevated. This gives molecular support for the reprogramming [19,63,69,92].

While the ceiling culture approach has been useful in defining the morphological change from lipid-laden adipocytes to fibroblast-like DFAT cells, the underlying processes, notably the function of liposecretion vs. lipolysis, require further study. It has been proven that lipid droplets are secreted rather than merely lipolyzed during dedifferentiation, and this active liposecretion process is driven by the microenvironmental conditions in ceiling culture. Recently, breakthroughs combining live-imaging and culture chips have identified dynamic actin-driven lipid droplet secretions as a mechanical mechanism that is crucial for dedifferentiation [30,93]. Importantly, the process is not a cell culture artifact, but a pathophysiologically associated phenomenon supported by in vivo evidence. Crucially, adipocyte dedifferentiation is a responsive biological program that occurs under specific physiological and pathological conditions, rather than being merely an experimental artifact [92]. The duality of this plasticity is a central theme: it serves adaptive, homeostatic functions in physiology, but it can become maladaptive, driving disease progression in pathology [68]. Under physiological conditions, such as in the mammary gland during pregnancy and lactation, mature adipocytes can undergo lipid loss and dedifferentiate into preadipocyte-like cells. These cells are subsequently redifferentiate into adipocyte during involution, contributing to tissue remodeling [92]. Similarly, in the dermal adipose tissue of the skin, adipocyte dedifferentiation and redifferentiation occur in synchrony with hair follicle cycling, supporting the evidence of its role in normal tissue homeostasis and regeneration [68]. However, this same plasticity can be co-opted in disease. In pathological conditions like fibrosis and cancer, adipocytes undergo a distinct process often termed adipocyte–mesenchymal transition (AMT), transdifferentiating into myofibroblasts, or cancer-associated fibroblasts (CAFs) [60,68]. This transition is a main contributor to excessive ECM deposition, tissue stiffening, and tumor progression. This suggests that adipocyte dedifferentiation may occur under mechanical pressure and extreme conditions [19].

The dual function of adipocyte dedifferentiation is a crucial finding. It can be adaptive, as indicated in wound healing, where dedifferentiated adipocytes transform into fibroblasts that facilitate tissue repair and collagen deposition, accelerating scarless healing [19,94,95]. This reparative function is further supported by evidence that DFAT cells can contribute to the regeneration of various tissues, including cardiac muscle after infarction, smooth muscle in the bladder and urethra, and neural tissue [25]. However, it can also be maladaptive, causing insulin resistance and interrupting normal adipogenic activity, which leads to metabolic dysregulation; PPARG signaling deficits are associated with this dysfunction. This metabolic dysfunction is exacerbated in chronic conditions like obesity, in which hypertrophic adipocytes become dysfunctional, promoting a pro-inflammatory and pro-fibrotic microenvironment that further drives pathological dedifferentiation and fibrosis [96].

Most remarkably, this review highlights the novel and detrimental role of dedifferentiation in the tumor microenvironment, where adipocytes dedifferentiate into myofibroblast-like cells through mechanotransduction. The dedifferentiated adipocytes may increase cancer aggressiveness and promote fibrosis, especially through Wingless/Integrated/β-catenin (Wnt/β-catenin) signaling [16,24,68,92]. Signaling pathways such as TGF-β, Wnt, and inflammatory cytokines are central drivers of this pathological transition [60]. TGF-β1 is a potent inducer, stimulating SMAD2/3 phosphorylation and upregulating collagen gene expression during the dedifferentiation of human adipocytes, thereby linking this pathway directly to fibrotic transformation [97].

A biological underpinning of these various functional outcomes is provided by the concomitant transcriptome alterations, which include the overexpression of stemness, proliferation, migratory markers, and the downregulation of adipogenic genes (e.g., PPARG and C/EBPα). This molecular shift underpins the therapeutic potential of DFAT cells. However, critical knowledge gaps still exist, especially with regard to the precise in vivo triggers and the individual signaling pathways that control the shift between deleterious (fibrosis/cancer) and beneficial (repair) dedifferentiation [67]. The major challenge for clinical translation lies in this very duality. The primary risk of harnessing adipocyte dedifferentiation is the potential to inadvertently activate the same pathological pathways, such as fibrosis, metabolic dysfunction, or tumor support, that this review outlines as detrimental [98]. Therefore, future therapeutic strategies must precisely control the process, ensuring that dedifferentiated cells are directed toward regenerative fates while safeguarding against uncontrolled proliferation or fibrotic transformation. Future research should be conducted to identify specific markers for adipocyte-derived fibroblasts in vivo and explore the therapeutic potential of modulating their plasticity, either by promoting it for regenerative medicine applications, like scarless wound healing, or by inhibiting it to overcome metabolic diseases or cancer progression. To specifically address the critical need to disentangle beneficial from deleterious dedifferentiation in vivo, we propose three concrete experimental directions: First, the development of more advanced, intersectional lineage-tracing strategies is essential. Employing dual recombinase systems (e.g., Adipoq-CreERT2 combined with a fibroblast-specific Flp driver) would allow for the precise, simultaneous labeling of mature adipocytes and their fibroblastic progeny within the same tissue. This approach could unequivocally identify “transition cells” and track their ultimate fate—whether they redifferentiate into adipocytes (a reversible, beneficial outcome) or commit to a pro-fibrotic myofibroblast lineage (a maladaptive outcome) in models of wound healing versus fibrosis [92]. Second, leveraging spatiotemporal single-cell multi-omics across contrasting in vivo contexts would reveal the decisive molecular switches. Performing single-cell RNA sequencing coupled with spatial transcriptomics on tissues undergoing physiological remodeling (e.g., mammary gland involution) and pathological progression (e.g., bleomycin-induced fibrosis or tumor growth) would map distinct transcriptional trajectories and niche signals that steer dedifferentiated cells toward regenerative or pathogenic fates [17]. Third, integrating mechanobiology into in vivo models is crucial, given the potent role of physical cues. Implanting engineered biomaterial scaffolds of defined stiffness into adipose depots, or using controllable compression devices in animal models, would allow researchers to directly test how mechanical forces—acting through pathways like Wnt/β-catenin—bias dedifferentiated adipocytes toward a tumor-promoting, fibrotic phenotype versus a reparative one [24]. Together, these targeted approaches would move the field beyond observation toward a mechanistic understanding that is necessary for therapeutic intervention.

## 5. Conclusions

Mature adipocyte dedifferentiation represents a fundamental and dynamic form of cellular plasticity with significant, yet context-dependent, roles in physiology and disease. This review synthesizes evidence for its dual function: beneficial in contexts like wound healing but detrimental in fibrosis, metabolic disease, and cancer. However, this synthesis must be contextualized within key limitations of the current evidence base, including a heavy reliance on in vitro ceiling culture models, heterogeneity across animal studies, and a notable scarcity of direct human clinical data.

These constraints help delineate realistic translational pathways. Near-term applications are most feasible in diagnostics and ex vivo cell therapy, such as developing plasticity-associated biomarkers or using patient-derived DFAT cells for engineered tissue repair. In contrast, systemic pharmacological interventions aimed at modulating this plasticity in vivo remain speculative, given the pleiotropic roles of core pathways like TGF-β and Wnt in both health and disease.

Therefore, future research must prioritize bridging these gaps. A critical next step is to identify the precise in vivo regulators, niche signals, and fate-commitment markers that decisively steer adipocyte plasticity toward regenerative versus pathological outcomes. Achieving this will require cross-species validation and human tissue mapping to translate this compelling biological mechanism into safe and effective therapeutic strategies.

## Figures and Tables

**Table 1:** Subcategories of 15 articles mentioning “Dedifferentiation of Mature Adipocytes”.

Subcategory	References
Cancer (*n* = 6)	[19,20,21,22,23,24]
Morphology/Cell Culture Techniques (*n* = 3)	[10,25,26]
Obesity/Metabolism (*n* = 2)	[27,28]
Genetic (*n* = 2)	[17,29]
Myofibroblast (Scar Repair/Skin Inflammation) (*n* = 2)	[30,31]

## Data Availability

No new data were created or analyzed in this study, Data sharing is not applicable to this article.

## Supplementary Materials

The following supporting information can be downloaded at https://www.mdpi.com/article/10.3390/biomedicines14010095/s1, Table S1: PRISMA 2020 Checklist.

## Abbreviations

The following abbreviations are used in this manuscript:

Ad/N1ICD	Adenovirus expressing the Notch1 intracellular domain
Adipoq-Cre	Adiponectin promoter-driver Cre-recombinase
ADIPOQ	Adipocyte Q
ADSCs	Adipose-derived stem cells
AT	Adipose tissue
BAT	Brown adipose tissue
BrdU	Bromodeoxyuridine
CAAs	Cancer-associated adipocytes
C/EBPα	C CAAT/Enhancer-Binding Protein Alpha
C/EBPβ	C CAAT/Enhancer-Binding Protein Beta
DFAT	Dedifferentiated Fat
ECs	Endothelial cells
FABP4	Fatty Acid-Binding Protein 4
FASN	Fatty Acid Synthase
LDs	Lipid Droplets
LIPE	Lipase Hormone-Sensitive
LPL	Lipoprotein Lipase
LPS	Liposarcoma
Mia-PaCa2	Human pancreatic carcinoma cell line
MSCs	Mesenchymal stem cells
PDK4	Pyruvate dehydrogenase kinase 4
PPARG	Peroxisome Proliferator-Activated Receptor Gamma
SEM	Scanning Electron Microscopy
SREBF1	Sterol Regulatory Element-Binding Transcription Factor 1
SVF	Stromal vascular fraction
TEM	Transmission Electron Microscopy
TGFβ1	Transforming Growth Factor Beta 1
WAT	White Adipose Tissue
Wnt/β-catenin	Wingless/Integrated/β-catenin
α-SMA	Alpha-smooth muscle actin
