# Supplementary material for: Dedifferentiation of Mature Adipocytes and Their Future Potential for Regenerative Medicine Applications
Source: Biomedicines. 2026 Jan 2;14(1):95. doi: 10.3390/biomedicines14010095 (PMC12838972; doi:10.3390/biomedicines14010095)
Supplement: Supplementary file 1 [file biomedicines-14-00095-s001.zip › biomedicines-4034134-supplementary.pdf]

PRISMA 2020 Checklist

Section and Topic	Item #	Checklist item	Reported on page #
**TITLE**			
Title	1	Identify the report as a systematic review.	Page 1, Line 1
**ABSTRACT**			
Abstract	2	See the PRISMA 2020 for Abstracts checklist.	Page 1, Line 15-20
**INTRODUCTION**			
Rationale	3	Describe the rationale for the review in the context of existing knowledge.	Page 2-3, Line 75-106 and Figure 1
Objectives	4	Provide an explicit statement of the objective(s) or question(s) the review addresses.	Page 3, Line 106-114
**METHODS**			
Eligibility criteria	5	Specify the inclusion and exclusion criteria for the review and how studies were grouped for the syntheses.	Page 4, Line 129-140
Information sources	6	Specify all databases, registers, websites, organisations, reference lists and other sources searched or consulted to identify studies. Specify the date when each source was last searched or consulted.	Page 4, Line 126-129, Table S1
Search strategy	7	Present the full search strategies for all databases, registers and websites, including any filters and limits used.	Page 4, Line 126-136
Selection process	8	Specify the methods used to decide whether a study met the inclusion criteria of the review, including how many reviewers screened each record and each report retrieved, whether they worked independently, and if applicable, details of automation tools used in the process.	Page 4, Line 134-138
Data collection process	9	Specify the methods used to collect data from reports, including how many reviewers collected data from each report, whether they worked independently, any processes for obtaining or confirming data from study investigators, and if applicable, details of automation tools used in the process.	Page 4, Line 143-144
Data items	10a	List and define all outcomes for which data were sought. Specify whether all results that were compatible with each outcome domain in each study were sought (e.g. for all measures, time points, analyses), and if not, the methods used to decide which results to collect.	Page 4, Line 142-145 and Figure 2
	10b	List and define all other variables for which data were sought (e.g. participant and intervention characteristics, funding sources). Describe any assumptions made about any missing or unclear information.	Page 4, Line 143-144
Study risk of bias assessment	11	Specify the methods used to assess risk of bias in the included studies, including details of the tool(s) used, how many reviewers assessed each study and whether they worked independently, and if applicable, details of automation tools used in the process.	Page 4, Line 143-144
Effect measures	12	Specify for each outcome the effect measure(s) (e.g. risk ratio, mean difference) used in the synthesis or presentation of results.	N/A
Synthesis methods	13a	Describe the processes used to decide which studies were eligible for each synthesis (e.g. tabulating the study intervention characteristics and comparing against the planned groups for each synthesis (item #5)).	Page 4, Line 126-143
	13b	Describe any methods required to prepare the data for presentation or synthesis, such as handling of missing summary statistics, or data conversions.	N/A; No data transformation or statistical preparation was required.
	13c	Describe any methods used to tabulate or visually display results of individual studies and syntheses.	Page 4, Line 144-145 and Figure 2
	13d	Describe any methods used to synthesize results and provide a rationale for the choice(s). If meta-analysis was performed, describe the model(s), method(s) to identify the presence and extent of statistical heterogeneity, and software package(s) used.	Page 4, Line 142-145
	13e	Describe any methods used to explore possible causes of heterogeneity among study results (e.g. subgroup analysis, meta-regression).	N/A; Assessment of statistical heterogeneity

PRISMA 2020 Checklist

Section and Topic	Item #	Checklist item	Reported on page #
			was not applicable due to the absence of quantitative analysis.
	13f	Describe any sensitivity analyses conducted to assess robustness of the synthesized results.	N/A; No sensitivity analyses were conducted.
Reporting bias assessment	14	Describe any methods used to assess risk of bias due to missing results in a synthesis (arising from reporting biases).	Not reported; Reporting bias was not formally assessed due to the qualitative nature of the synthesis and inclusion of review articles only.
Certainty assessment	15	Describe any methods used to assess certainty (or confidence) in the body of evidence for an outcome.	Not reported; The certainty of evidence was not formally assessed, as this review was focused on mechanistic insights rather than effect estimation.
**RESULTS**			
Study selection	16a	Describe the results of the search and selection process, from the number of records identified in the search to the number of studies included in the review, ideally using a flow diagram.	Page 4-5-6, Line 150-187, Figure 2 and Figure 3; Table 1
	16b	Cite studies that might appear to meet the inclusion criteria, but which were excluded, and explain why they were excluded.	Page 5-6, Line 159-194 and Figure 3; Table 1
Study characteristics	17	Cite each included study and present its characteristics.	Page 5-6, Line 168-194 and Figure 3; Table 1
Risk of bias in studies	18	Present assessments of risk of bias for each included study.	N/A; Formal risk-of-bias assessments were not conducted, as the included studies were predominantly mechanistic in vitro and animal studies for which standardized risk-of-bias tools are not applicable.
Results of individual studies	19	For all outcomes, present, for each study: (a) summary statistics for each group (where appropriate) and (b) an effect estimate and its precision (e.g. confidence/credible interval), ideally using structured tables or plots.	N/A; Quantitative summary statistics and effect estimates were not applicable, as this review synthesizes qualitative findings from experimental studies. Results of individual

PRISMA 2020 Checklist

Section and Topic	Item #	Checklist item	Reported on page #
			studies are reported narratively.
Results of syntheses	20a	For each synthesis, briefly summarise the characteristics and risk of bias among contributing studies.	Page 6-14, Line 193-500 and Figure 4; Table 1 and Table 2.<br>For each thematic synthesis the characteristics of contributing studies are summarized narratively, including biological context and mechanistic focus. Formal risk of bias assessment was not performed.
	20b	Present results of all statistical syntheses conducted. If meta-analysis was done, present for each the summary estimate and its precision (e.g. confidence/credible interval) and measures of statistical heterogeneity. If comparing groups, describe the direction of the effect.	N/A; No statistical syntheses or meta-analyses were conducted.
	20c	Present results of all investigations of possible causes of heterogeneity among study results.	N/A; Investigations of statistical heterogeneity were not conducted, as no quantitative synthesis was performed.
	20d	Present results of all sensitivity analyses conducted to assess the robustness of the synthesized results.	N/A; Sensitivity analyses were not performed because no statistical synthesis was undertaken.
Reporting biases	21	Present assessments of risk of bias due to missing results (arising from reporting biases) for each synthesis assessed.	Not assessed, given the narrative and mechanistic nature of the included studies.
Certainty of evidence	22	Present assessments of certainty (or confidence) in the body of evidence for each outcome assessed.	N/A
**DISCUSSION**			
Discussion	23a	Provide a general interpretation of the results in the context of other evidence.	Page 14, Line 503-512
	23b	Discuss any limitations of the evidence included in the review.	Page 14, Line 514-516
	23c	Discuss any limitations of the review processes used.	Page 15, Line 565-573
	23d	Discuss implications of the results for practice, policy, and future research.	Page 15-16, Line 573-599
**OTHER INFORMATION**			

PRISMA 2020 Checklist

Section and Topic	Item #	Checklist item	Reported on page #
Registration and protocol	24a	Provide registration information for the review, including register name and registration number, or state that the review was not registered.	To be added to Page 4, Line 127, Registered to Open Science Framework Registration: **10.17605/OSF.IO/BTVAP**
	24b	Indicate where the review protocol can be accessed, or state that a protocol was not prepared.	To be added to Page 4, Line 127, Registered to Open Science Framework Registration: **10.17605/OSF.IO/BTVAP**
	24c	Describe and explain any amendments to information provided at registration or in the protocol.	N/A
Support	25	Describe sources of financial or non-financial support for the review, and the role of the funders or sponsors in the review.	Page 17, Line 626
Competing interests	26	Declare any competing interests of review authors.	Page 17, Line 630
Availability of data, code and other materials	27	Report which of the following are publicly available and where they can be found: template data collection forms; data extracted from included studies; data used for all analyses; analytic code; any other materials used in the review.	Page 17, Line 629

From: Page MJ, McKenzie JE, Bossuyt PM, Boutron I, Hoffmann TC, Mulrow CD, et al. The PRISMA 2020 statement: an updated guideline for reporting systematic reviews. BMJ 2021;372:n71. doi: 10.1136/bmj.n71

For more information, visit: http://www.prisma-statement.org/
